# Positive and negative aspects of the COVID-19 pandemic among a diverse sample of US adults: an exploratory mixed-methods analysis of online survey data

**DOI:** 10.1186/s12889-023-17491-w

**Published:** 2024-01-02

**Authors:** Stephanie A Ponce, Alexis Green, Paula D. Strassle, Anna María Nápoles

**Affiliations:** 1 Division of Intramural Research, National Institute on Minority Health and Health Disparities, Bethesda, MD USA; 2 Division of Intramural Research , National Institute on Minority Health and Health Disparities, 11545 Rockville Pike, 2 White Flint North, Room C13, Rockville, MD 20818 USA

**Keywords:** COVID-19 pandemic, Posttraumatic growth, Gender, Race-ethnicity, Age, Telework, Economic impact, Social impact, Relationships

## Abstract

**Background:**

The COVID-19 pandemic had a profound social and economic impact across the United States due to the lockdowns and consequent changes to everyday activities in social spaces.

**Methods:**

The COVID-19’s Unequal Racial Burden (CURB) survey was a nationally representative, online survey of 5,500 American Indian/Alaska Native, Asian, Black/African American, Latino (English- and Spanish-speaking), Native Hawaiian/Pacific Islander, White, and multiracial adults living in the U.S. For this analysis, we used data from the 1,931 participants who responded to the 6-month follow-up survey conducted between 8/16/2021-9/9/2021. As part of the follow-up survey, participants were asked “What was the worst thing about the pandemic that you experienced?” and “Was there anything positive in your life that resulted from the pandemic?” Verbatim responses were coded independently by two coders using open and axial coding techniques to identify salient themes, definitions of themes, and illustrative quotes, with reconciliation across coders. Chi-square tests were used to estimate the association between sociodemographics and salient themes.

**Results:**

Commonly reported negative themes among participants reflected disrupted lifestyle/routine (27.4%), not seeing family and friends (9.8%), and negative economic impacts (10.0%). Positive themes included improved relationships (16.9%), improved financial situation (10.1%), and positive employment changes (9.8%). Differences in themes were seen across race-ethnicity, gender, and age; for example, adults ≥ 65 years old, compared to adults 18–64, were more likely to report disrupted routine/lifestyle (37.6% vs. 24.2%, *p* < 0.001) as a negative aspect of the pandemic, and Spanish-speaking Latino adults were much more likely to report improved relationships compared to other racial-ethnic groups (31.1% vs. 14.8–18.6%, *p* = 0.03).

**Discussion:**

Positive and negative experiences during the COVID-19 pandemic varied widely and differed across race-ethnicity, gender, and age. Future public health interventions should work to mitigate negative social and economic impacts and facilitate posttraumatic growth associated with pandemics.

**Supplementary Information:**

The online version contains supplementary material available at 10.1186/s12889-023-17491-w.

## Introduction

The impact of the COVID-19 pandemic on morbidity and mortality, as well as the social, behavioral, and economic impacts have been well-documented. As of May 23, 2023, the CDC estimates that the COVID-19 pandemic has caused 6,152,982 hospitalizations and 1,128,903 deaths in the United States (U.S) [1]. In addition to the pandemic itself, mitigation measures adopted during the pandemic to reduce transmission across the U.S. also had major social and economic impacts [2]. Efforts to mitigate the spread of COVID-19 infection, including social distancing, lockdowns, and transitions to remote work, became a source of life disruptions and emotional distress [3, 4]. Racial-ethnic disparities in the burden of COVID-19 morbidity and mortality, as well as negative social and economic consequences have been reported.

However, mitigation efforts and COVID-related policies also may have had positive impacts. Traumatic events, life crises, and illnesses, although challenging to move through, have often resulted in positive psychological change as people seek new ways of making meaning from these experiences. These positive psychological changes that result from traumatic events have been referred to as posttraumatic growth and defined as “positive psychological changes experienced as a result of the struggle with trauma or highly challenging situations.” [5] Previous research has indicated that women experience higher levels of posttraumatic growth, compared to men, and that increasing age moderates posttraumatic growth among women [6]. During the COVID-19 pandemic, women have been found to report higher posttraumatic growth during quarantine [7] and COVID-19 related hospitalization [8]; however, these studies were not based in the United States.

Additionally, in response to the COVID-19 pandemic, many companies adapted by creating new telework and flexible work schedule policies during lockdowns, [9, 10] which have been associated with improved work-life balance and family relationships [11, 12]. Initial reports during the pandemic indicate that the lockdown period also served as a time to find oneself [13], pursue new passions and hobbies [13], and save money [14] due to the lack of regular social outings and a decrease in transportation related expenses such as gas. COVID-19 associated posttraumatic positive growth experiences have not been well-researched, especially across diverse racial-ethnic groups.

Given the extensive social, behavioral, and economic changes and mitigation measures resulting from the COVID-19 pandemic, it is important to understand the positive and negative perceived consequences of these events among U.S. adults, and any differences across race-ethnicity, gender, and age. Thus, the purpose of this mixed-methods study was to capture the voices of a diverse, national sample of American Indian/Alaska Native, Asian, Black/African American, Native Hawaiian/Pacific Islander, Latino (English- and Spanish-speaking), White, and multiracial adults (≥ 18 years old) living in the U.S and perceived major positive and negative impacts of the pandemic.

## Methods

### Study population and survey development

For this study we used data from the COVID-19’s Unequal Racial Burden (CURB) online survey, which was administered by YouGov, a nonpartisan consumer research firm based in Palo Alto, CA, which uses a proprietary, opt-in survey panel comprised of over 1.8 million US residents to conduct nationally representative surveys. Panel members are recruited through a variety of methods to ensure diversity, and then were matched to a theoretical target sample. For this study, the target sample was drawn from the 2018 American Community Survey 1-year sample data, and included 1,000 Asian, 1,000 Black/African American, 1,000 Latino (including 500 Spanish-speaking), 1,000 White, 500 American Indian/Alaska Native, 500 Native Hawaiian/Pacific Islander, and 500 multiracial adults aged ≥ 18 years (n = 5,500 total). The survey was conducted in both English and Spanish (Latino participants only). The baseline CURB survey was first created in English, translated into Spanish by an American Translators Association certified translator, and then finalized by four bilingual/bicultural researchers via reconciliation and decentering methods. Questions added or modified in the follow-up survey were translated into Spanish by our bilingual/bicultural researchers.

The baseline survey was completed between December 8, 2020, and February 17, 2021, and the 6-month follow-up survey between August 16, 2021, and September 9, 2021 (35.1% response rate), Supplemental Table 1. For this analysis, we included all participants who responded to both the baseline and 6-month follow-up survey (n = 1,931). The CURB survey development and sampling design details have been previously described [15].

The National Institutes of Health Office of Institutional Review Board Operations determined that this study does not qualify as human subjects research because data were de-identified (IRB# 000166).

### Identifying negative and positive aspects of the COVID-19 pandemic

As part of the 6-month follow-up survey, all participants were asked two open-ended questions: (1) What was the worst thing about the pandemic that you experienced? and (2) Was there anything positive in your life that resulted from the pandemic? Verbatim responses were coded independently using open and axial coding methods in two steps by two coders (SAP, AG) [16]. Responses from Spanish-speaking Latino participants were translated into English, reviewed, and reconciled by two bilingual-bicultural research team members (SAP, AMN) prior to coding. In the first step, coders categorized responses to both questions using open coding methods (breaking the data into discrete parts and creating codes to label them). Then, applying axial coding methods (i.e., codes were organized into categories/themes), the responses for each question were coded for salient themes, definitions of themes, and illustrative quotes. Consensus was reached through iterative meetings between the coders and the rest of the study team.

### Participant sociodemographics

Race-ethnicity was captured by asking respondents “Which one of the following would you say best describes your race/ethnicity?” with response options of Latino/a/x or Hispanic, American Indian or Alaska Native, Asian, Black or African American, Pacific Islander, White, and multiracial. Latino participants were further stratified into English- and Spanish-speaking, depending on their survey language preference; 68.9% of Spanish-speaking Latino participants also self-reported limited English proficiency, compared to only 5.4% of English-speaking Latino participants. Gender was categorized as male, female, and transgender or nonbinary. Age was captured as a continuous variable and categorized as 18–34, 35–49, 50–64, and ≥ 65 years old.

### Statistical analysis

Descriptive statistics and Chi-square tests were used to compare the prevalence of salient themes for the two open-ended questions across self-reported race-ethnicity, gender, and age. Due to the small number of participants that identified as transgender or non-binary (n = 26), comparisons across gender were restricted to comparing male and female participants only. All analyses were conducted using SAS version 9.4 (SAS Inc., Cary, NC). Due to the relatively low response rate of the follow-up survey, results were not weighted to generate nationally representative estimates.

## Results

### Worst aspects of the COVID-19 pandemic

Overall, 1,511 of 1,931 participants (78.2%) responded to “What was the worst thing about the pandemic that you experienced?” Respondent characteristics can be found in Supplemental Table 2.

**Fig. 1:**
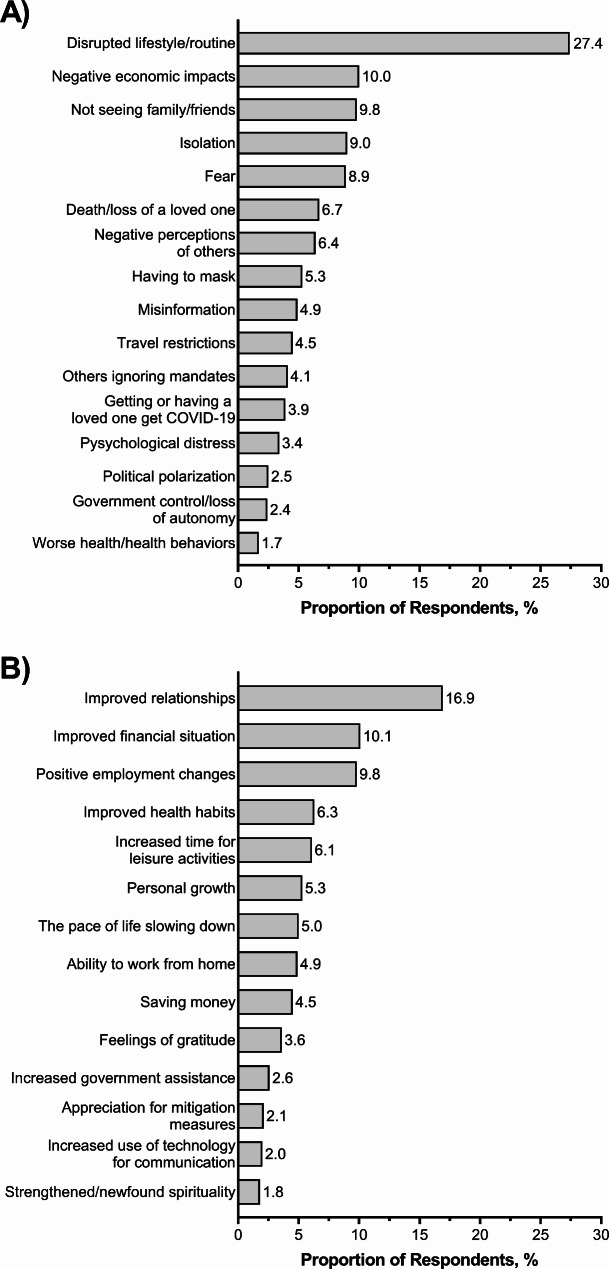
Overall prevalence of reported (A) negative and (B) positive impacts of the COVID-19 pandemic among a diverse sample of adults living in the US, COVID-19’s Unequal Racial Burden (CURB) survey, 8/16/2021-9/9/2021

Salient themes, definitions, and illustrative quotes for the worst aspects of the COVID-19 pandemic are reported in Table 1. Overall, the most commonly reported negative aspect was *disrupted lifestyle/routine* (27.4%), Fig. 1A. Responses included *disrupted lifestyle/routine* due to reduced services and restrictions in places like school, healthcare settings, restaurants, places of worship, and other places of recreation or routine outings. Missed important life events such as graduations, weddings, and internship opportunities were also noted. Adults ≥ 65 years-old, compared to adults 18–64, were more likely to report *disrupted routine/lifestyle* (37.6% vs. 24.2%, *p* < 0.001) as a negative impact of the pandemic, Supplemental Fig. 1A. No significant racial-ethnic or gender differences in the prevalence of *disrupted lifestyle/routine* were observed.

**Table 1:** Salient themes, definitions, and illustrative quotes based on open-ended responses to the question “What was the worst thing about the pandemic that you experienced?”, COVID-19’s Unequal Racial Burden (CURB) survey, 8/16/2021-9/9/2021

Theme	Definition	Illustrative Quotes
Disrupted lifestyle/routine	Changes in regular routines, social activities, school activities, household or caretaking responsibilities, dining out, concerts or other entertainment, religious services, missed or delayed milestone events (graduation, retirement, wedding); restrictions on use of public spaces; boredom, nothing to do; staying home, confinement; new safety measures such as masking, business restrictions, social distancing.	“Lockdown with the kids, unable to go to school in person.” “Not being able to dine in at restaurants.” “Not being able to attend Mass, especially during Holy Week and Easter.”
Negative economic impacts	Personal, nationwide, or statewide negative economic experiences, such as inflation, increased prices, reduced income, loss of equity, loss of income/savings/investments, greater financial insecurity, shortages of goods and services.	“Everything costs more now, food, gas, merchandise, homes, etc…” “Inability to find certain groceries” “Salary cuts and freezes while prices are rising”
Not seeing family/friends	Not seeing/not spending time/or not visiting friends, family members, acquaintances; missing family and friends.	“Not being able to hang out with family and friends sometimes”
Isolation	Feelings of loneliness and being isolated; inability to socialize, have social interactions; being away or distant from others.	“Isolation from everyone”
Fear	Generalized fear, worry; specific fears or anxieties, such as fear of getting COVID-19 or transmitting the virus, being afraid “to go anywhere”; uncertainty or feeling of never-ending pandemic; fear due to a sense of loss of normalcy.	“Fear that this will never end” “Feeling worried about family members”
Death/loss in general or of a loved one	Mention of loss/death in general; loss/death of a family member, friend, acquaintance.	“Death of loved ones that I never got to say goodbye to so there was no closure” “Watching people die.”
Negative perceptions of others	Feelings of animosity towards others, negative words used to describe intelligence, capacity, character of others or community (often due to differing opinions and practices related to COVID-19 safety measures, political opinions regarding mandates; personal or observed experiences of discrimination or violence because of race (Asian or other), perceived increase in racism since the pandemic.	“People have become more angry, impatient and confrontational.” “How selfish people are.” “People being racist towards me because I’m Asian”
Having to mask	Mask mandates/requirements by local/state/federal governments and businesses	“Having to wear a useless mask to grocery shop.”
Misinformation	Lack of consistent information and communication, misinformation, perceived lies and confusion surrounding information or news.	“The lies from governments” “The media hiding the real numbers if they would have told the truth we never would have been locked down.” “Untrue sensationalized news reporting, I don’t watch the news anymore.”
Travel restrictions	Cancelled/delayed travel, feeling uncomfortable traveling, suspension of international travel and vacations.	“Could not travel abroad to visit family and friends”
Others ignoring mandates	Discomfort with others not following COVID-19 related mandates (masking, vaxxing, social distancing, etc.).	“Frustration with people who will not mask or vaccinate”
Getting or having a loved one get COVID-19	Self, loved one, friend, or acquaintance contracting COVID, getting sick; experiencing symptoms associated with COVID-19 infection.	“The symptoms of COVID19, body aches”
Psychological distress	Experiences of anxiety, depression, stress, mental fatigue.	“My depression getting worse.” “Lots of stress and anxiety.”
Political polarization	Polarization along political party lines, perceived poor government response, opposing views/ideologies and disagreement over handling of the COVID-19 pandemic.	“The politics of COVID and the division of people over beliefs.”
Government control//loss of autonomy	Government infringement on one’s rights and freedoms, imposition of laws/mandates/restrictions; perceptions that government responses to the pandemic were excessive and not necessary.	“Ignorant, unhelpful government policy.” “Loss of freedom and destruction of the economy”
Worse health/health behaviors	Non-specific illnesses or symptoms; changes in weight or exercise frequency; increases in risky behaviors such as smoking and drinking.	“Weight gain due to being stuck inside” “My health got worse. I exercised less, gained weight, stress ate, and there were other stressors that caused worse mental health/anxiety, too, like stress of getting sick, family stress (helping with child care/caretaking), etc. All that stress and no healthy outlet, [inability] to go to the gym was one of the worst parts of the pandemic.”

*“My senior year of college was ruined by the pandemic. I missed my senior cross-country season and the chance to go to nationals in indoor track.”* (Female, 18–34 years old, White).

The next most commonly reported worst aspect was *negative economic impacts* (10.0%), Fig. 1A. Respondents reported negative changes in employment/unemployment, decreased financial security, decreased job security, increased cost of products/living, and increased shortages. Younger adults (< 65 years old) were more likely to report *negative economic impacts* compared to adults ≥ 65 years old (11.0% vs 2.3%, *p* = 0.001), Supplemental Fig. 1B.

*“Not being able to purchase certain food items, toilet paper, paper towels, masks, disinfectant spray. The prices of items increasing.”* (Female, 18–34 years old, multiracial).

*“Recently having to live off one income plus whatever unemployment pays. My bank account has been negative, often because of bills being pulled and not having the funds.”* (Female, 35–49 years old, English-speaking Latino).

**Fig. 2:**
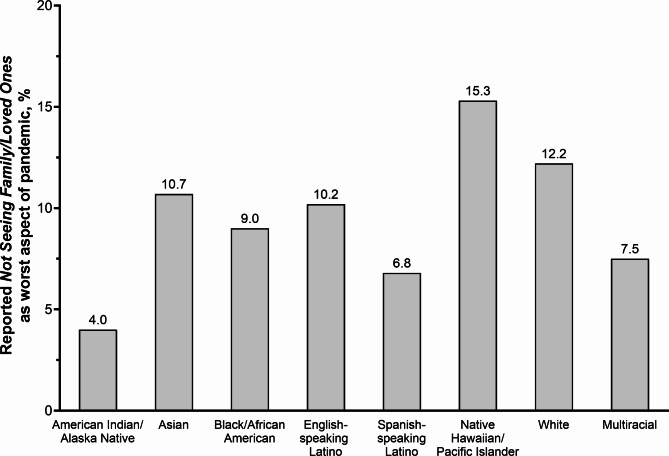
Prevalence of reporting *not seeing family/loved ones* during the COVID-19 pandemic, stratified by race-ethnicity, among a diverse sample of adults living in the US, COVID-19’s Unequal Racial Burden (CURB) survey, 8/16/2021-9/9/2021

Overall, *not seeing family/loved ones* (9.9%) and (9.0%) *isolation* were reported as negative aspects by roughly 1 in 10 respondents, respectively, Fig. 1A. Reasons included concerns of transmission and decreased quality of social interactions due to masking and social distancing. Women, compared to men, were more likely to report *not seeing family/loved ones* as the worst aspect of the pandemic (12.5% vs. 6.6%, *p* < 0.001). Increasing age was associated with being more likely to report *not seeing family/loved ones* (18–34 years old, 6.8%; 35–49 years old, 8.5%; 50–64 years old, 11.0%;and ≥ 65 years old, 13.1%; *p* = 0.034). Racial-ethnic differences were also observed (*p* = 0.004), with Native Hawaiian/Pacific Islander (15.3%), White (12.2%), Asian (10.7%), and English-speaking Latino (10.2%) adults reporting higher prevalence of *not seeing family/loved ones* compared to, Fig. 2. No significant differences in the reporting of *isolation* were seen across race-ethnicity, gender, or age.

*" Me senti alejado del mundo y necesitaba contacto con otras personas pero creo que la pandemia todavia no ha pasado” (“I felt cut off from the world and needed contact with other people but I believe that the pandemic has not yet passed”)* (Female, 50–64 years old, Spanish-speaking Latino).

*“Being separated from my kids and grandchildren who live in another state 2500 miles from my home. During the pandemic we planned and canceled three trips to see them. Finally made the trip after 18 months without in person contact.”* (Male, ≥ 65 years old, White).

Other negative aspects reported were *fear* (8.9%), *death/loss of a loved one* (6.7%), *negative perceptions of others* (6.4%), *having to mask* (5.3%), *misinformation* (4.9%), *travel restrictions* (4.5%), *others ignoring mandates* (4.1%), *getting or having a loved one get COVID-19* (3.7%), *psychological distress* (3.4%), *political polarization* (2.5%), *government control/loss of autonomy* (2.4%), and *worse health/health behaviors* (1.7%), Fig. 1A.

### Positive aspects of the COVID-19 pandemic

Overall, 1,033 participants responded to “*Was there anything positive in your life that resulted from the pandemic?*” (53.5%). Respondent characteristics are reported in Supplemental Table 2.

**Fig. 3:**
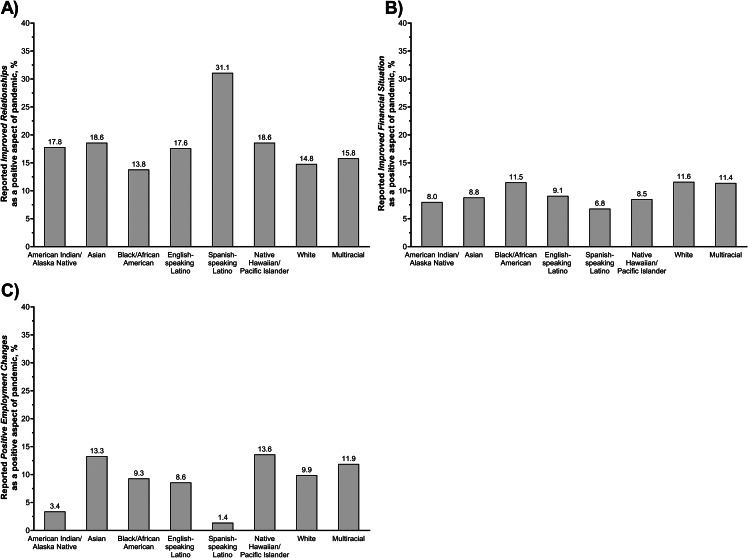
Prevalence of reporting (A) *improved relationships*, (B) *improved financial situations*, and (C) *positive employment changes*, stratified by race-ethnicity, among a diverse sample of adults living in the US, COVID-19’s Unequal Racial Burden (CURB) survey, 8/16/2021-9/9/2021

Salient themes, definitions, and illustrative quotes for the positive impacts of the COVID-19 pandemic are reported in Table 2. The most commonly reported positive impact of the COVID-19 pandemic was *improved relationships* (16.9%), Fig. 1B. Reasons for *improved relationships* included spending more time at home and more quality time with loved ones. Women (18.8% vs. 15.0% *p* = 0.03) and adults 18–49 years old (20.1% vs. 14.2%, *p* = 0.004) were more likely to report improved relationships, compared to men and older age groups, Supplemental Fig. 2A. Spanish-speaking Latino adults were also much more likely to report improved relationships compared to other racial-ethnic groups, (31.1% vs. 14.8–18.6%, *p* = 0.03), Fig. 3A.

**Table 2:** Salient themes, definitions, and illustrative quotes based on open-ended responses to the question “Was there anything positive in your life that resulted from the pandemic?”, COVID-19’s Unequal Racial Burden (CURB) survey, 8/16/2021-9/9/2021

Theme	Definition	Illustrative Quotes
Improved relationships	Increased contact, quality time, value placed on relationships with partner, family member, friends, pet, neighborhood, community; improved interpersonal communication.	“Got to spend more time with family. Reconnected with friends I had lost touch with.”
Improved financial situation	Increased financial security due to increased income, receiving government assistance (ex. public stimulus funding, food stamps), increased savings, decreased spending.	“I received unemployment and extra federal UI [unemployment insurance] benefits so was able to save and payoff my auto loan early.” “I’m a little more ahead financially.”
Positive employment changes	Improved work, job, employment conditions for self or partner; job stability as a result of the pandemic.	“I didn’t lose job since I am a healthcare worker” “I got a job I love! I am working for the first time in many years.” “I got paid more money from my current employer.”
Improved health and healthy habits	Better health, improved strength, weight loss, less substance use, improved diet quality, increased physical activity, e.g., walking, running; not getting sick, not getting COVID.	“Exercised more” “Focused more on my own fitness” “Good health” “I ate a lot better than I had previously and also exercised regularly.”
Increased time for leisure and home improvement activities	More time for hobbies, acquiring new skills and knowledge, playing an instrument, reading, woodworking, cleaning/housework, home improvement projects.	“Was able to do a lot of house projects!” “Yes, started checking things off my bucket list-kayaking, jet skiing, snorkeling with manatees, skydiving indoors.”
Personal growth	New sense of being in touch with one’s emotions, learning what is important, learning about and caring for oneself, newfound perspective on value of life and others, pursuing learning opportunities, improved psychosocial wellbeing.	“Did some internal reflection” “I got to love myself more”
Adopting a slower pace of life	Resting, sleeping, relaxing, slowing down, spending more time at home/indoors, more time to focus on self, less stress.	“I could stay home more without feeling guilty for ignoring relationships.” “I slowed down, not a lot of running around”
Ability to work from home	Positive impact of increased job flexibility and ability to work from home.	“Being able to work from home”
Saving money	Decreased spending, increased savings.	“Spending more time with my family and getting to spend less money on entertainment and food.”
Feelings of gratitude	New appreciation for life and getting through tough times, being grateful/thankful for what is important in life.	“I learned to appreciate life more and enjoy everything I could when I had the chance.”
Increased government assistance	Received assistance from the government in form of stimulus funding, food stamps, unemployment benefits, loan deferment, pandemic electronic benefit transfer or P-EBT (public program to replace food benefits lost at child care/school).	“Government stimulus REALLY helped. That money was a God send. Also extra food stamps helped”
Appreciation of mitigation measures	Increased use of masks, improved personal hygiene such as washing of hands, improved cleanliness when handling groceries, mail, etc.	“Due to the nature of my work, I must have face-to-face contact with the public and I would always catch colds throughout the year. But, because everyone was masked and kept their distance, I have not caught a cold in over a year. Caught Covid but not a single cold to date. So, I guess that counts as a positive.” “didn’t have to shake hands, reject hugs, explain that I don’t like public displays of affection even among friends.”
Increased use of technology for communication	Increased use of Zoom, FaceTime, virtual communication media to stay in touch, reconnect long distance, attend school; more time talking on the phone.	“I have spoken more on the phone with elderly relatives which we all appreciate” “I spent a lot of time FaceTiming with my nieces.”
Strengthened/newfound spirituality	Improved relationships with God, religion, church, religious figures; more time devoted to spiritual practices.	“I spend more time listening to scriptures.” “I have been spending more time focusing on my faith.”

*“Got to spend more time with family. Reconnected with friends I had lost touch with.”* (Male, 35–49 years old, Asian).

*Improved financial situation* (10.1%) and *positive employment changes* (9.8%) were also reported as positive results of the pandemic. Reasons included increased savings, increased work flexibility, increased income, and reduced commutes. *Improved financial situation* was more commonly reported by men compared to women (12.3% vs. 8.2%, *p* = 0.003), Supplemental Fig. 2B. *Positive employment changes* were more often reported by younger adults (18–49 years old) compared to older age groups (13.5% vs. 6.8%, *p* < 0.001), Supplemental Fig. 2C. American Indian/Alaska Native and Spanish-speaking Latino adults were less likely to report *improved financial situation* and *positive employment changes* as positive aspects of the pandemic, compared to other racial-ethnic groups (11.2% v. 6.4%, *p* = 0.001), Fig. 3B,C.

*“I received unemployment and extra federal UI benefits so was able to save and payoff my auto loan early.”* (Female, 50–64 years old, Black/African American).

*“I got paid more money from my current employer.”* (Male, 18–34 years old, multiracial).

Participants also reported *improved health habits* (6.3%), *increased time for leisure activities* (6.1%), *personal growth* (5.3%), *pace of life slowing down* (5.0%), *ability to work from home (4.9%), saving money* (4.5%), *feelings of gratitude* (3.6%), *increased government assistance* (2.6%), *appreciation for mitigation measures* (2.1%), *increased use of technology for communication* (2.0), and *strengthened/newfound spirituality* (1.8%), Fig. 1B.

## Discussion

This mixed-methods study investigated perceived negative and positive experiences associated with the COVID-19 pandemic in from the perspectives of racially-ethnically diverse U.S. adults. Overall, negative aspects of the pandemic were reported more frequently than positive ones. Salient perceptions of the worst aspects of the pandemic included disrupted lifestyle/routine, negative economic impacts, not seeing family/loved ones, and isolation. Disrupted lifestyles and routines and not seeing family and loved ones were more frequently reported by older adults compared to younger age groups, while younger adults were more likely to report negative economic impacts. Not seeing family and loved ones was more frequently reported also by Native Hawaiian/Pacific Islander, White, Asian, and English-speaking Latino adults than other racial-ethnic groups. The most common positive aspects of the pandemic reported included improved relationships, improved financial situation, and positive employment changes. Women, adults aged 18–49 years, and Spanish-speaking Latino adults were more likely than men, older age groups, and other racial-ethnic groups to report improved relationships with family and friends as positive aspects. American Indian/Alaska Native adults and Spanish-speaking Latino adults, were less likely to report positive economic impacts, compared to other racial-ethnic groups. These findings highlight the importance of qualitative research in capturing life experiences during the COVID-19 pandemic.

The high prevalence of disrupted lifestyles can be attributed to restrictions on public spaces, the transition to online spaces, and changes in available services during the peak of the pandemic in the U.S. are expected. New York, for example, closed all nonessential businesses and restricted out-of-home activities for residents beginning in March 22, 2020 [17]. However, in the United States these policies were implemented on a state-by-state (and sometimes city-by-city) basis, leading to differences across the country [18]. Many of these closures/restrictions led to students and employees transitioning their classroom and office spaces to home settings, significantly changing their household dynamic. Learning and work environments were dependent on the spaces available, which in some cases were crowded, loud, and suboptimal, especially for those in a household with more than one person distance learning or working from home [19]. Routine outings for groceries and necessities also became high-risk and, in some cases, contactless, further reducing opportunities for movement and social interaction [20]. Reduced opportunities for social interaction and movement during the pandemic have been reflected in reported decreases in steps and increases in screentime [21].

Disruptions in daily activities, business closures, and restricted out-of-home activities also appeared to have severe negative consequences on individual and household finances. Individuals reported increased financial insecurity, loss of job or job security, and an overall negative change in their financial situation because of the pandemic. Both our study and others have found that younger adults disproportionately experienced financial strain because of the pandemic [22, 23]. Other studies have also reported that financial hardship during the COVID-19 pandemic was more substantial among racial-ethnic minorities and low-income adults [24, 25].

Interestingly, we also found that two of the most common positive aspects of the COVID-19 pandemic were improvements in financial situations and employment situations. The COVID-19 pandemic resulted in large increases in the proportion of the workforce teleworking and most likely led to improved work life balance due to fewer commute hours. Over half of individuals able to work from home have reported that working from home has made it easier to balance work with their personal life [26]. Beyond work from home flexibility, opportunities for hazard pay, government pandemic assistance, and reduced spending were associated with the pandemic allowing for some households and individuals to strengthen or gain financial security. However, increased financial stability has been primarily reported by upper-income and middle-income households, retired adults, and among adults with a bachelor’s degree or higher [25, 27].

Improvements in relationships with family, loved ones, and neighbors were also reported, most likely due to increased use of immediate social networks to satisfy social interaction needs and reduce isolation, when broader engagement was limited due to enforced restrictions on public spaces and travel. Lockdowns across the country led to spending more time at home with family and loved ones [28]. Conversely, studies have also found an increase in reported loneliness and isolation as a result of the COVID-19 pandemic and lockdown, [29–31] prompting the U.S. Surgeon General to release a 2023 report on the “loneliness and isolation” epidemic in the U.S., exacerbated by the pandemic [32]. Quarantining during prior outbreaks has been associated with poor mental health, and increased feelings of isolation, anger, confusion, and frustration [33, 34]. Additional research on the impact of social networks and household structures on feelings of isolation and loneliness during lockdowns and quarantines are needed to better understand how to prevent poor mental health during future outbreaks.

Social distancing also encouraged increased use of texting, video calling, and social media as means of communication [35]. It could be that increased use of these alternatives to in-person contact led to more frequent and/or improved quality of social interactions and relationships for some individuals. For example, in a study among Italian adults, voice and video calls, online gaming platforms, social media, and watching movies in party mode were associated with reduced feelings of loneliness, anger, and boredom due to perceived social support from these interactions [36, 37]. A study among Australian adults found that communicative smartphone use during the pandemic was found to increase friendship satisfaction over time [38]. Further research in the potential of technology to reduce isolation and improve social connections is warranted. However, cultural values influence interpersonal interaction styles and preferences and need to be considered. For example, the high proportion of Spanish-speaking Latino adults reporting improved relationships may be tied to familism (i.e., the cultural emphasis on one’s family as a main source of emotional and instrumental social support when needed, including elements of loyalty, reciprocity, and solidarity within one’s family) [39]. At least one other study has also found that Latino adults reported positive changes in their relationships during the pandemic [40].

Similar to findings from the SARS epidemic in 2002–2004, [41] our participants also reported positive experiences of personal growth, living life at a slower pace, more time for leisure activities, and increased feelings of gratitude due to the pandemic. The additional time to pause and reflect, paired with the hardships of the pandemic, led many to adopt new perspectives characterized by greater appreciation of the people in their lives and for life in general [42]. Our study results support the important role of gratitude and its potential for bolstering resilience during times of tragedy and uncertainty [43]. In total, these experiences can be framed as posttraumatic growth resulting from the challenges of the pandemic. Such personal growth may serve a protective role in buffering the negative effects of fear associated with pandemics, and enhance satisfaction with life [44] and psychological wellbeing especially among older adults [45, 46]. Public health efforts to support such growth during widespread pandemics could focus on evidence-based methods for generating posttraumatic growth and emotional resiliency, such as education to enhance emotional regulation, disclosure, meaning finding in trauma, and service to others [5].

This study adds to the sparse literature characterizing social patterning of positive and negative changes during the COVID-19 pandemic, including the potentially contradictory effects of the pandemic on individuals. Our study’s use of qualitative methods has allowed us to capture the nuances of respondents’ lived experiences within the context of the COVID-19 pandemic. Differences across age, gender, and race-ethnicity captured in this study point to a diversity of experiences and the need for recovery strategies tailored to community needs. The nationally representative sample employed in our study also facilitated our capturing a broad range of experiences across racial/ethnic minority, language, age, and gender groups.

Study limitations include our low response rate to the follow-up survey, which may have inhibited our ability to detect statistically significant differences in responses to the two open-ended questions. Response rates also differed across participant race-ethnicity and not all participants responded to the open-ended questions, which may have impacted findings. Additionally, our 6-month follow-up survey was conducted in August-September 2021, and respondents’ experiences may have changed over time as the pandemic continued to evolve. The surveys were also administered online, and individuals with limited internet access or familiarity with technology and limited literacy may have been less likely to participate. For example, we did not find racial-ethnic differences in reports of negative economic experiences, possibly due to the online nature of our survey, which may have biased our sample toward those with greater economic resiliency. However, the potential of online surveys to conduct qualitative research has been previously noted [47]. Furthermore, the CURB survey was only administered in English and Spanish (for Latino participants), which means adults who prefer other languages were likely to be excluded. This may have especially impacted our Asian adult cohort, as 31.9% of Asian adults nationally are estimated to have limited English proficiency (compared to 12.3% in our survey). Finally, our survey was not designed to capture an equal number of individuals living in each state or city, and the effects of the heterogeneous COVID-19 responses across the country may not have been captured fully.

## Conclusion

In this study, we used a mixed-methods approach to capture the perceived negative and positive impacts of the COVID-19 pandemic on people’s lives among a diverse group of adults. Responses varied by race-ethnicity, gender, and age, indicating that the lived experiences of diverse groups differed, most likely due to variation in access to economic and social resources for managing COVID-related disruptions and hardships. A more nuanced understanding of such variation in the perceived positive and negative effects of pandemics can inform tailored public health efforts to mitigate potentially harmful factors and support posttraumatic growth and emotional resiliency during health crises.

### Electronic supplementary material

Below is the link to the electronic supplementary material.

Supplementary Material 1

## Data Availability

Data is available upon reasonable request. Contact Dr. Paula Strassle (paula.strassle@nih.gov) for access.

## Abbreviations

CURB: COVID-19’s Unequal Racial Burden
U.S: United States
CDC: Centers of Disease Control and Prevention
SAP: Stephanie A. Ponce
AG: Alexis Green
