# Supplementary material for: Positive and negative aspects of the COVID-19 pandemic among a diverse sample of US adults: an exploratory mixed-methods analysis of online survey data
Source: BMC Public Health. 2024 Jan 2;24:22. doi: 10.1186/s12889-023-17491-w (PMC10762906; doi:10.1186/s12889-023-17491-w)

**Supplemental Materials**

**Supplemental Table 1.** Response rates to the follow-up survey, overall and stratified by race-ethnicity, COVID-19’s Unequal Racial Burden (CURB) survey, 8/16/2021-9/9/2021.

|  | **N** | **Response**  **Rate (%)** |
| --- | --- | --- |
| **Overall** | **1,931** | **35.1** |
| American Indian/Alaska Native | 174 | 34.8 |
| Asian | 430 | 43.0 |
| Black/African American | 312 | 31.2 |
| Native Hawaiian/Pacific Islander | 59 | 11.8 |
| Latino | 261 | 26.1 |
| Spanish-speaking | 74 | 14.9 |
| English-speaking | 187 | 37.3 |
| White | 493 | 49.3 |
| Multiracial | 202 | 40.4 |

**Supplemental Table 2.** Sociodemographic characteristics of participants who answered two open-ended questions about negative and positive aspects of the pandemic, COVID-19’s Unequal Racial Burden (CURB) survey, 8/16/2021-9/9/2021.

|  | *Overall* | *What was the worst thing about the pandemic that you experienced?* |  | *Was there anything positive in your life that resulted from the pandemic?* |
| --- | --- | --- | --- | --- |
|  | **N (%)** | **N (%)** | | **N (%)** |
| **Overall** | 1931 (100) | 1511 (78.2) | | 1033 (53.5) |
| **Race/ethnicity** |  |  | |  |
| American Indian/Alaska Native | 174 (9.7) | 137 (9.1) | | 75 (7.3) |
| Asian | 430 (22.3) | 321 (21.2) | | 256 (24.8) |
| Black/African American | 312 (16.2) | 220 (14.6) | | 172 (16.7) |
| Latino |  |  | |  |
| *English-speaking ^a^* | 187 (9.7) | 145 (9.6) | | 96 (9.3) |
| *Spanish-speaking ^a^* | 74 (3.8) | 60 (4.0) | | 50 (4.8) |
| Native Hawaiian/Pacific Islander | 59 (3.1) | 49 (3.2) | | 39 (3.8) |
| Multiracial | 202 (10.5) | 160 (10.6) | | 112 (10.8) |
| White | 493 (25.5) | 419 (27.7) | | 233 (22.6) |
| **Age group** |  |  | |  |
| 18-34 | 339 (17.6) | 248 (16.4) | | 190 (18.4) |
| 35-49 | 543 (28.1) | 404 (26.7) | | 320 (31.0) |
| 50-64 | 836 (43.3) | 682 (45.1) | | 421 (40.8) |
| ≥65 | 213 (11.0) | 177 (11.7) | | 102 (9.9) |
| **Gender** |  |  | |  |
| Female | 1025 (53.8) | 814 (53.9) | | 562 (54.5) |
| Male | 879 (46.2) | 677(44.8) | | 456 (44.2) |
| Transgender or non-binary^b^ | 26 (1.4) | 20 (1.3) | | 14 (1.4) |
| **Highest education level** |  |  | |  |
| Less than high school graduate | 72 (3.7) | 44 (2.9) | | 24 (2.3) |
| High school/GED | 419 (21.7) | 290 (19.2) | | 163 (15.8) |
| Some college/vocational school | 615 (31.9) | 486 (32.2) | | 311 (30.1) |
| College degree or higher**^c^** | 823 (42.7) | 691 (45.7) | | 535 (51.8) |
| **Family annual income^d^** |  |  | |  |
| <$20,000 | 260 (15.4) | 183 (13.7) | | 110 (12.0) |
| $20,000-$59,999 | 644 (38.0) | 507 (37.8) | | 324 (35.3) |
| $60,000-$99,999 | 400 (23.6) | 323 (24.1) | | 235 (25.6) |
| ≥$100,000 | 389 (23.0) | 328 (24.5) | | 249 (27.1) |
| Prefer not to say | 238 | 170 | | 115 |
| ^a^ Latino participants were stratified based on survey language preference  ^b^ Nonbinary includes individuals who reported being nonbinary, gender fluid, gender queer, 'other', and no gender  ^c^ Includes bachelor's degree, master's degree, and doctoral or postgraduate education  ^d^ Collected by YouGov at enrollment into panel and updated every 6 months | | | | |

**Supplemental Figure 1.** Prevalence of reporting A) *disrupted lifestyle* and B) *negative economic impacts* stratified by age, among a diverse sample of adults living in the US, COVID-19’s Unequal Racial Burden (CURB) survey, 8/16/2021-9/9/2021.

**
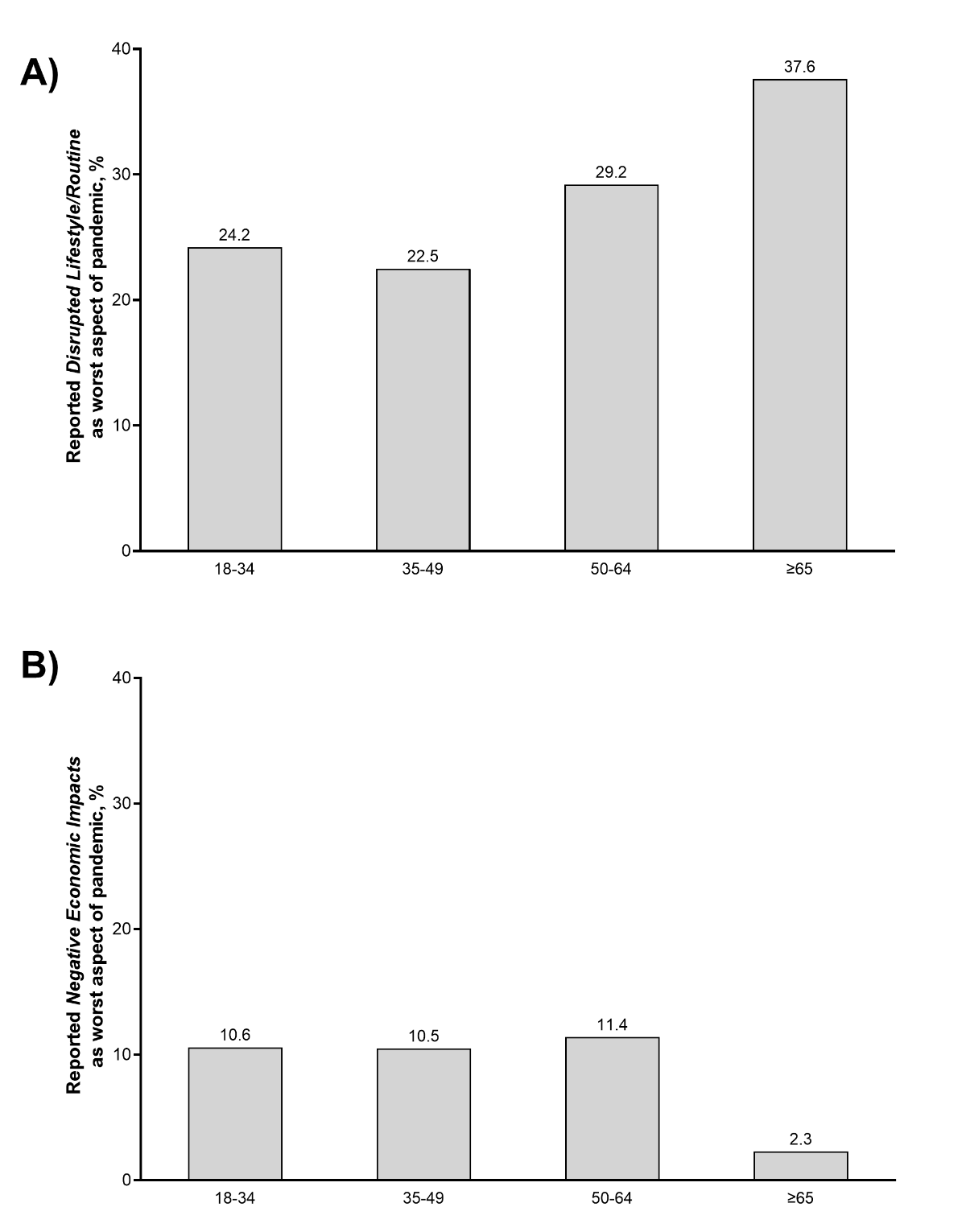
**

**Supplemental Figure 2.** Prevalence of reporting A) *improved relationships* stratified by gender, B) *improved financial situation* stratified by gender, and C) *positive employment changes* stratified by age, among a diverse sample of adults living in the US, COVID-19’s Unequal Racial Burden (CURB) survey, 8/16/2021-9/9/2021.


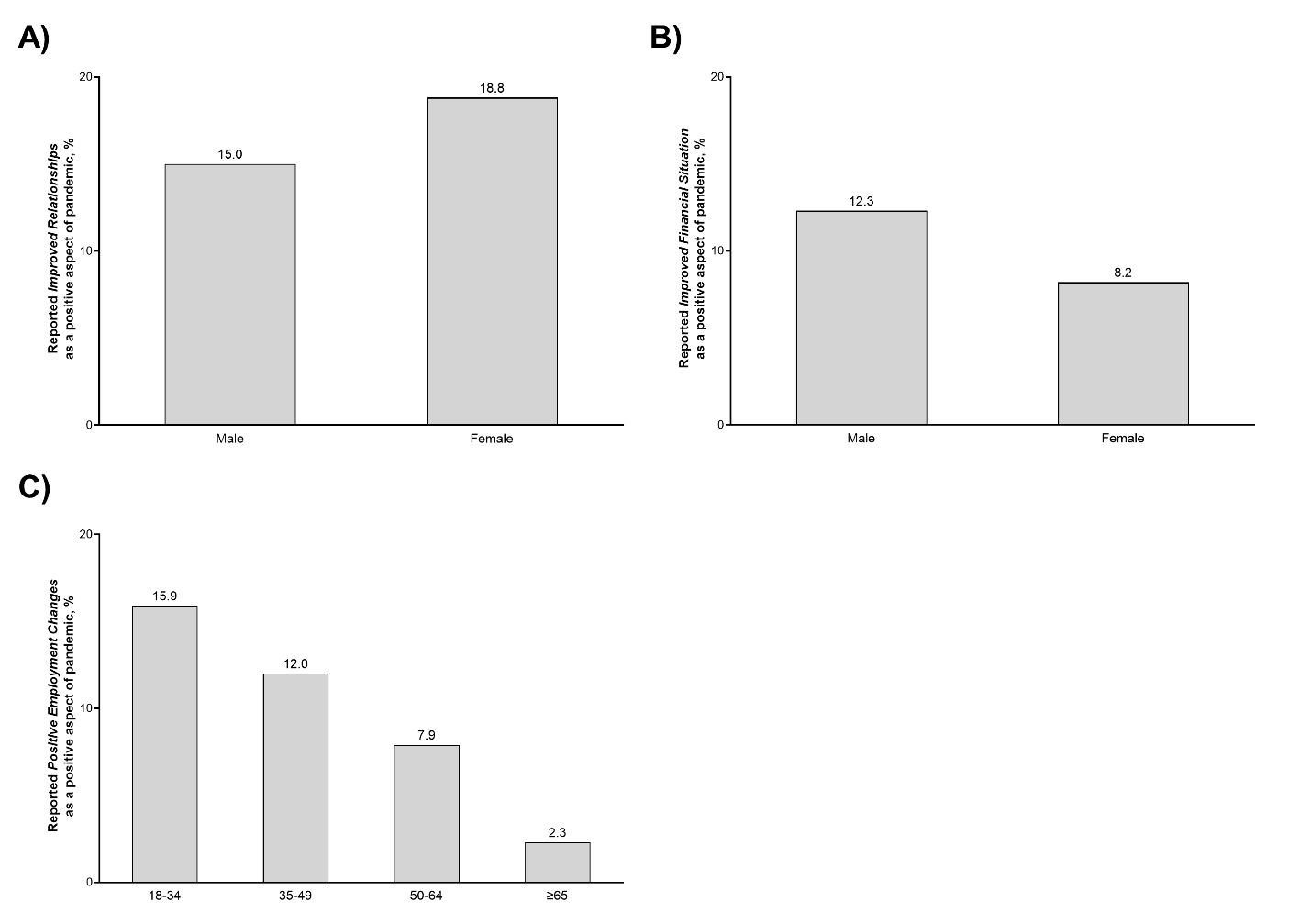

Supplement: Supplementary file 1 — Supplementary Material 1 [file 12889_2023_17491_MOESM1_ESM.docx]
